# Supplementary material for: Composition-Regulated Photocatalytic Activity of ZnIn2S4@CdS Hybrids for Efficient Dye Degradation and H2O2 Evolution
Source: Molecules. 2024 Aug 14;29(16):3857. doi: 10.3390/molecules29163857 (PMC11357647; doi:10.3390/molecules29163857)
Supplement: Supplementary file 1 [file molecules-29-03857-s001.zip › molecules-3131055-supplementary.pdf]

Supplementary Information

Composition-Regulated Photocatalytic Activity of $ZnIn_2S_4$@CdS Hybrids for Efficient Dye Degradation and H_2O_2 Evolution

Nikolaos Karamoschos [a], Andreas Katsamitros [a], Labrini Sygellou [b], Konstantinos S. Andrikopoulos [b,c], Dimitrios Tasis [a,d,*]

[a] *Department of Chemistry, University of Ioannina, 45110 Ioannina, Greece*

[b] *Foundation of Research and Technology Hellas, Institute of Chemical Engineering Sciences (ICE-HT), P.O. Box 1414, 26504 Rio Patras, Greece*

[c] *Department of Physics, University of Patras, Patras, 26504, Greece*

[d] *University Research Center of Ioannina (URCI), Institute of Materials Science and Computing, Ioannina 45110, Greece*

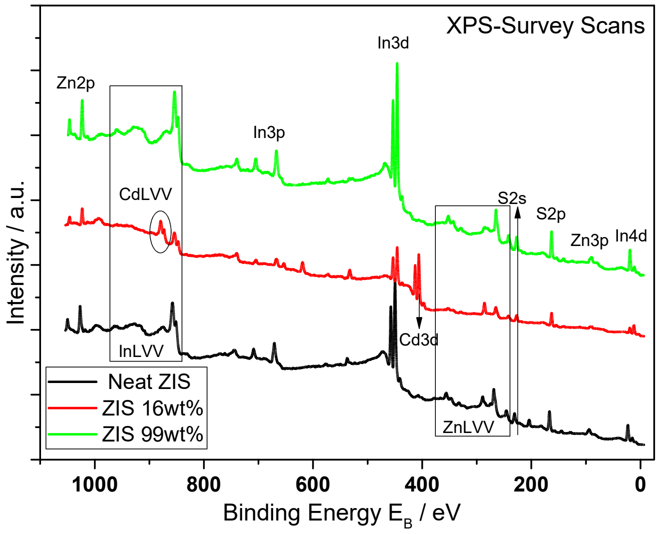

Figure S1. XPS Survey Scans from Neat ZIS, "ZIS 16 wt%" and "ZIS 99 wt%" samples.

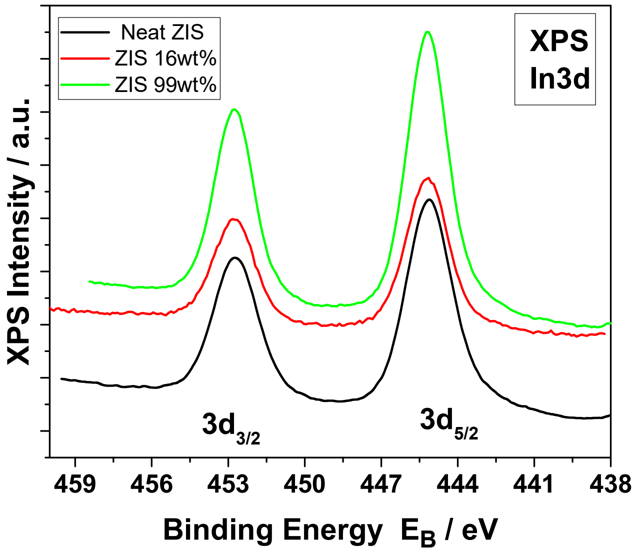

Figure S2. In3d XPS core level peaks of neat ZIS, "ZIS 16 wt%" and "ZIS 99 wt%" samples.

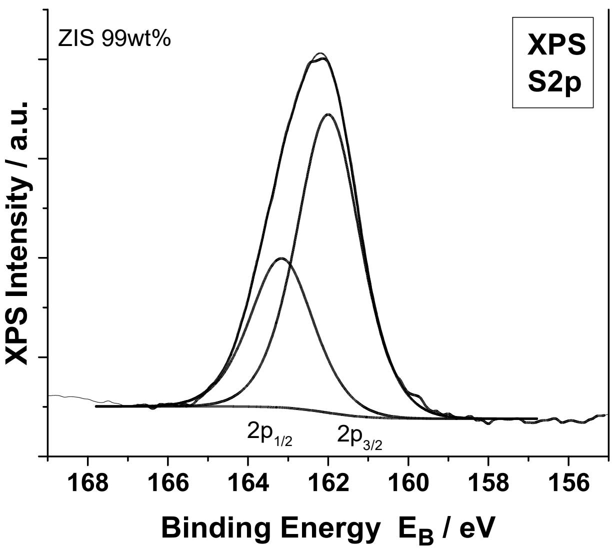

Figure S3. S2p XPS core level peaks of “ZIS 99 wt%” sample.

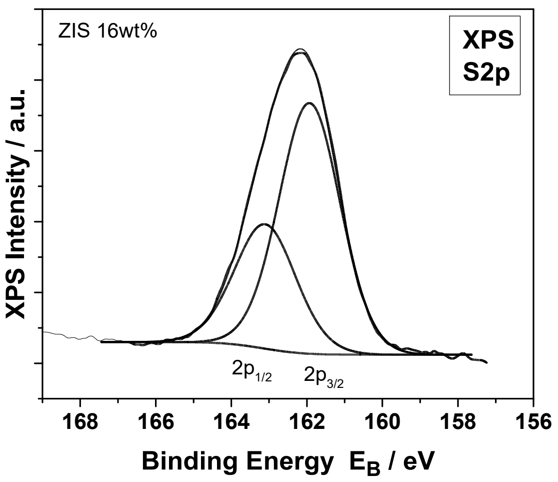

Figure S4. S2p XPS core level peaks of ZIS 16 wt% sample.

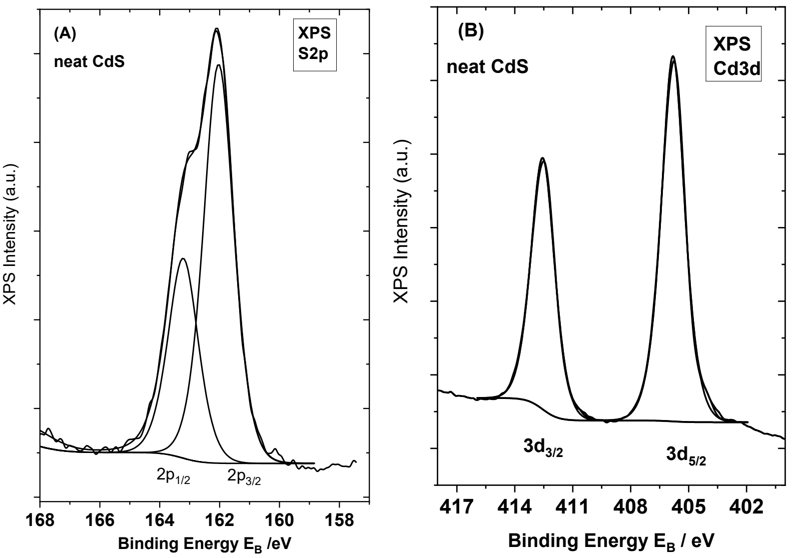

Figure S5. (A) S2p and (B) Cd3d XPS peaks of neat CdS sample

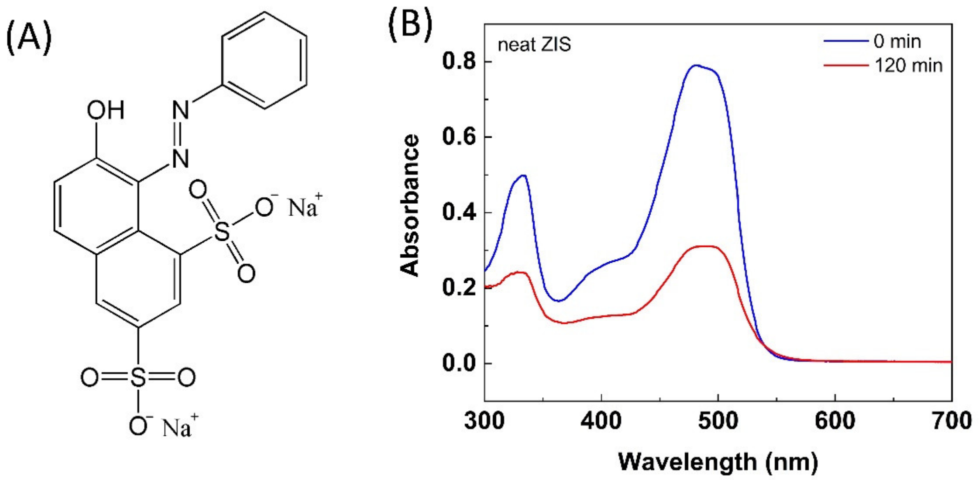

Figure S6. (A) Chemical formula of Orange G dye; (B) Evolution of dye absorption spectrum before and after irradiation.

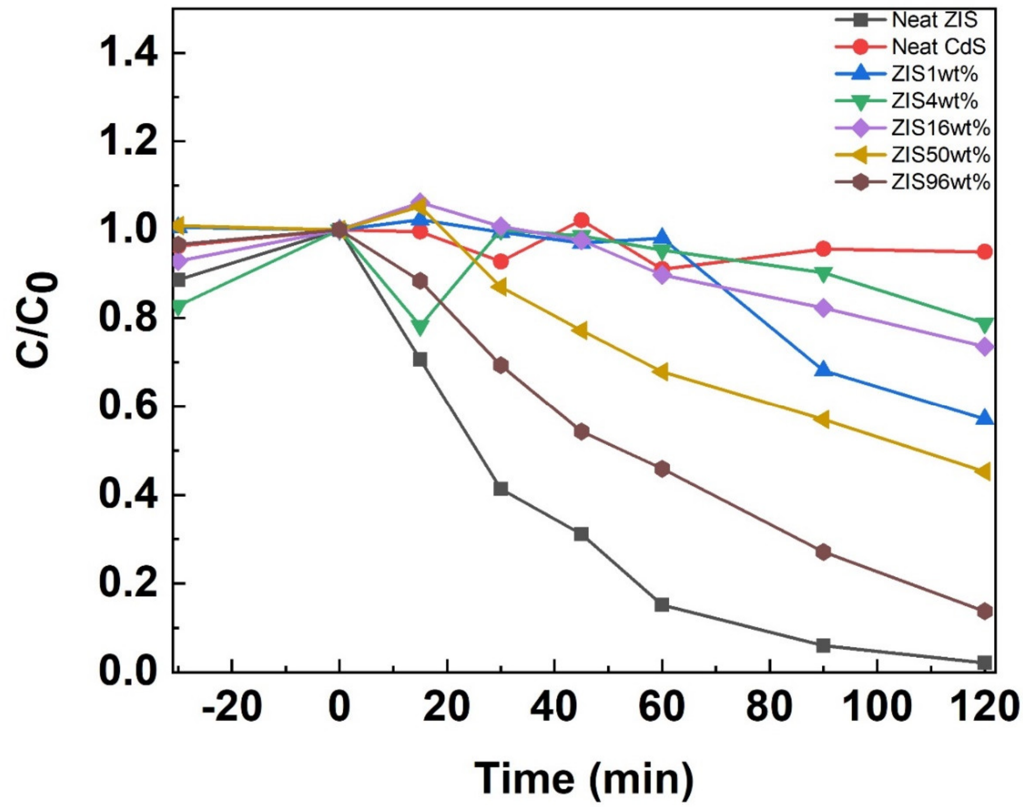

Figure S7. Dye decay kinetic profiles of studied samples.

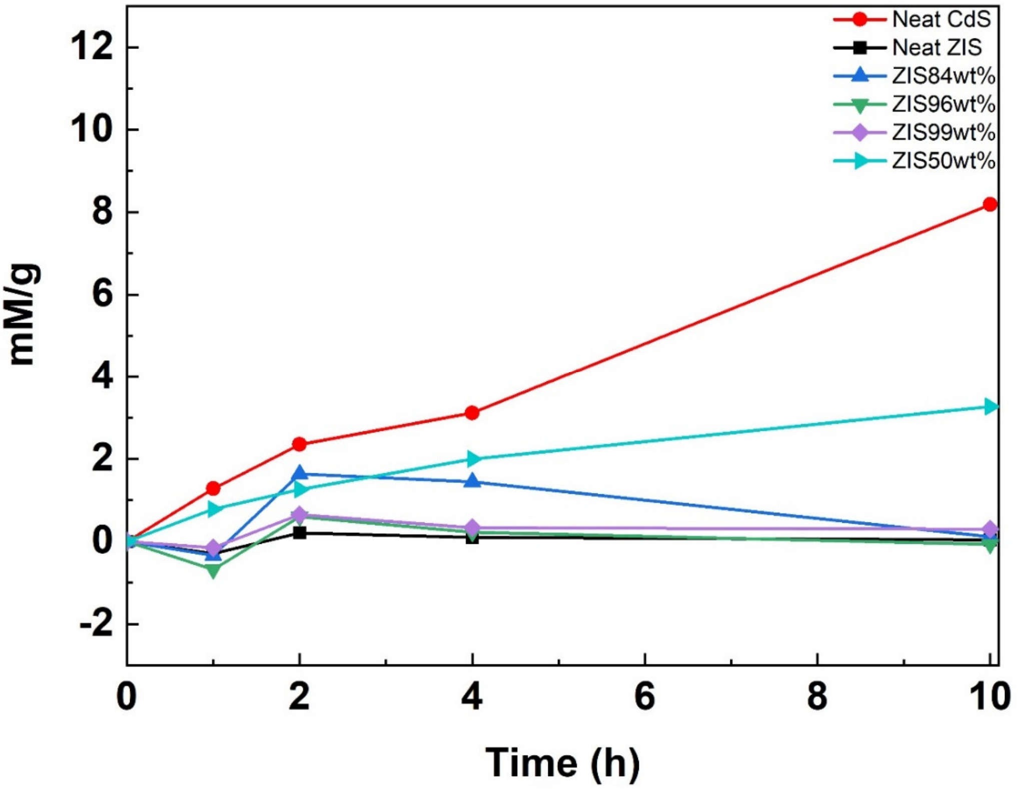

Figure S8. Kinetic profiles for H_2O_2 evolution of the studied samples.

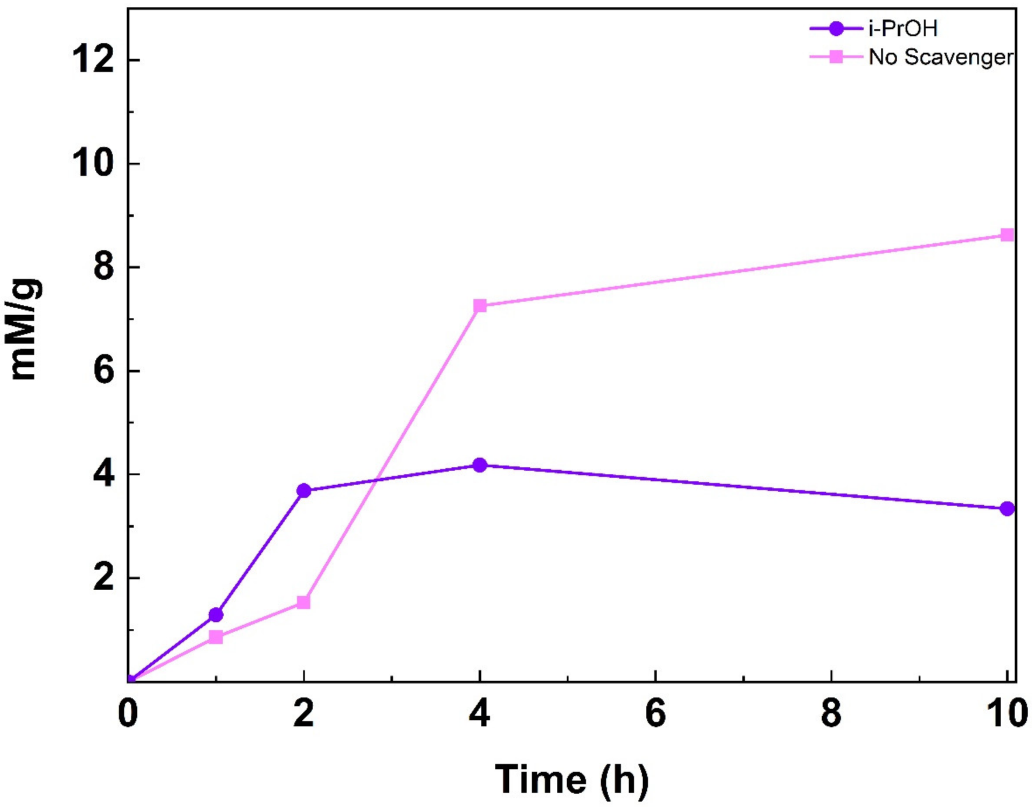

Figure S9. Scavenging experiments for H_2O_2 evolution experiments in the presence of "ZIS 16 wt%" sample.

Table S1. Atomic concentration (%) of neat ZIS, “ZIS 16 wt%” and “ZIS 99 wt%” samples, derived from the XPS results. The nominal % atomic concentration of neat ZIS is given in parentheses.

	In	Zn	S	Cd
Neat ZIS	30.6 (28.6)	14.5(14.3)	54.9(57.1)	-
ZIS 16wt%	15.3	8.0	53.5	23.2
ZIS 99wt%	28.7	14.0	57.1	0.3

Table S2. Literature data about degradation of organic substances and H_2O_2 evolution by ZIS-based hybrids

**ZIS-based hybrids**	**Organics degradation, (t_{50}, min)**	**H_2O_2 evolution in mM/g/h**	**Ref**
P-doped h-BN/ZIS	-	6	19
O-doped C_3N_4/ZIS	CTC antibiotic (35)	14	21
MOF/ZIS	2-nitrophenol (35)	-	22
g-C_3N_4/ZIS	-	16	23
TiO_2/ZIS	Tetracycline (20)	30	24
P-CoNi/ZIS	-	11	25
MOF@ZIS/CdS	2,4,5-dichlorophenol (90)	-	32
CdS/ZIS	-	6	33
CdS/ZIS	LEV antibiotic (40)	-	35
CdS/ZIS	Tetracycline (15)	60	36
MoO_3/ZIS	RhB dye (25)	-	38
CdS/ZIS	Orange G dye (20)	2	**This work**
